# Toxoplasma Serology Status and Risk of Miscarriage, A Case-Control Study among Women with A History of Spontaneous Abortion

**DOI:** 10.22074/ijfs.2019.5740

**Published:** 2019-07-14

**Authors:** Farnaz Kheirandish, Behrouz Ezatpour, Shirzad Fallahi, Mohammad Javad Tarahi, Pardis Hosseini, Arian Karimi Rouzbahani, Seyyed Javad Seyyed Tabaei, Soheila Akbari

**Affiliations:** 1 Razi Herbal Medicines Research Center, Department of Parasitology and Mycology, School of Medicine, Lorestan University of Medical Sciences, Khorramabad, Iran; 2 Department of Epidemiology and Biostatistics, School of Health, Isfahan University of Medical Sciences, Isfahan, Iran; 3 Student Research Committee, Lorestan University of Medical Sciences, Khorramabad, Iran; 4 Department of Parasitology and Mycology, School of Medicine, Shahid Beheshti University of Medical Sciences, Tehran, Iran; 5 Department of Obstetrics and Gynecology, School of Medicine, Lorestan University of Medical Sciences, Khorramabad, Iran

**Keywords:** Abortion, IgG Avidity, Pregnancy, Serology, Toxoplasmosis

## Abstract

**Background:**

*Toxoplasma gondii* is one of the major causes of abortion in pregnant women. Most cases of abortion occur in the acute phase of infection and early pregnancy. The purpose of this study was to investigate the association between spontaneous abortion and seropositive status of toxoplasmosis in women with first-time spontaneous abor- tion.

**Materials and Methods:**

This research is a case-control study on 240 serum samples from women experiencing spon- taneous abortion for the first time as the case group, and 240 serum samples from women who had a normal delivery with no history of abortion as the control group. The level of anti-*Toxoplasma gondii* IgM and IgG antibodies were assessed in serum samples using ELISA. To separate the acute and chronic infections, all IgM-positive samples in both groups and IgG-positive samples of the case group were examined using IgG avidity.

**Results:**

The *Toxoplasma* IgM antibody was detected in 3.3% (8/240) of the case group and 0.4% (1/240) of the con- trol group, which was a statistically significant difference between the two groups [P=0.019, odds ratio (OR)=10.266]. Of all samples 47.5% and 46.3% of the case and control groups were positive for *Toxoplasma* IgG antibody, respec- tively. Seven out of 8 (87.5%) IgM-positive serum samples from the case group had low IgG avidity, indicating acute infections, whereas all IgG-positive sera and 1 IgM-positive serum, which was related to the control group, showed a high IgG avidity, indicating chronic infections.

**Conclusion:**

Maternal acute toxoplasmosis during pregnancy is raised as one of the factors that increase the chance of spontaneous abortion. The necessary health training, especially on the parasite transmission ways to women before marriage, as well as the serological test in women before and during pregnancy is recommended. Polymerase chain reaction (PCR) and IgG avidity assays should be performed in the medical diagnostic laboratories for accurate distin- guishing of the initial infection of toxoplasmosis in the pregnant women.

## Introduction

Pregnancy is one of the most critical steps in women's lives, particularly those who want to become a mother for the first time. Abortion is a problem that any women might experience during pregnancy, and therefore suffer from psychological issues and medical expenses, which make it particularly important. One of the reasons for abortion is toxoplasmosis, which is due to an infection caused by *Toxoplasma gondii*, an obligate intracellular parasite, belonging to the phylum of Sporozoa, causing toxoplasmosis disease in humans and most of the warm- blooded animals around the world (1). This parasite, as one of the common human and animal pathogens, has accounted for numerous studies (2, 3). In humans, it is one of the most prevalent parasites, as in the serological studies it is estimated that nearly one-third of the human populations in Europe, South America, Africa, and Asia are infected with this parasite (4). Prevalence of *T. gondii* infection in pregnant women is investigated in different parts of the world, and estimated to be 14-77% (5). 

In general, a human typically becomes infected by three principal routes of transmission including drinking contaminated water or eating contaminated food, such as the tissue cyst in half-cooked contaminated meat or food that is contaminated with oocysts excreted from cat feces, and congenital transition, that means the transmission from an infected mother to her fetus (5). Toxoplasmosis infection might be acute or chronic with or without symptoms. The symptoms and complications of the disease mainly occur in the acute phase of infection. Following activation of the host immune system, the parasite proliferation is controlled and tissue cysts are formed in the host neuro-muscular tissues (3, 5). Although the acquired toxoplasmosis causes asymptomatic mild infections in people with a healthy immune system, it can cause severe clinical signs and even death in those with a weak or impaired immune systems. On the other hand, in people who are suffering from immune deficiency or are consuming immune-suppressive drugs, the chronic infection may be reactivated, causing severe and deadly complications such as encephalitis, myocarditis, and pneumonia (5). Transplacental transmission of *T. gondii* occurs mainly in the course of the first pregnancy (6). Congenital toxoplasmosis, which occurs during pregnancy, can cause spontaneous abortion, stillbirth, and some degrees of mental or physical retardation, hydrocephalus, blindness, and deafness (6, 7). Frequency and severity of the congenital toxoplasmosis are associated with the gestational age. The highest rate of congenital toxoplasmosis occurs in the third trimester of pregnancy, however, the highest infection severity is observed in the first and second trimesters, which can cause abortion or stillbirth (5-7). 

The global estimated incidence rate of congenital toxoplasmosis is 190,100 cases annually, with an approximate incidence rate of 1.5 cases per 1000 live births [95% confidence interval (CI): 179,300-206,300] (8). The previous studies in Iran have shown that the seroprevalence rates of toxoplasmosis among childbearing age women are totally 39.9% among childbearing age women (9) and 39.3% (95% CI ¼ 33.0-45.7%) among the general population (10). Infection is more prevalent in hot and humid areas and relatively rare in cold and dry areas. The prevalence of infection is different among various ethnic groups, but the difference is more related to genetic differences, environmental health, and cooking habits (11).

One of the most popular medical concerns around the world is how to diagnose acute congenital infections in a pregnant woman that may lead to spontaneous abortion. This type of abortion is the disposal of pregnancy products before the twentieth week of gestation, without the use of medical and mechanical factors (12). Serological tests are the common diagnostic methods for congenital toxoplasmosis (13). Enzyme-linked immunosorbent assay (ELISA) test is currently the most widespread and most commonly used serological diagnosis method for toxoplasmosis (14). In recent years, efforts have been made to improve the ability to diagnose infections in pregnant women and congenital infections in the fetus and newborn. There are already a number of new methods to prove that there is great value for this purpose. For example, IgG avidity and polymerase chain reaction (PCR) applied on body fluids and tissue, as well as the western blot technique on the mother and infant serum samples, can be mentioned (15).

With regard to geographical and climatic differences in the prevalence of toxoplasmosis, and the lack of sufficient and precise data on the role of the parasite in abortion, in this study, the seroprevalence of anti-*T. gondii* IgM and IgG antibodies were investigated in women with first abortion experience in Khorramabad, Lorestan province, Western Iran. In order to determine the acute and chronic infections, all IgM- and IgG-positive serum samples were evaluated using IgG avidity.

## Materials and Methods

### Study region

Lorestan province is the thirteenth province in Iran in terms of population and is considered as one of the most populous provinces in Iran. The city of Khorramabad is the capital of the province. Lorestan province is located in Western Iran and placed between the latitudes 32. 30´ and 48.1´ N and longitudes 55. 17´ and 61. 15´E. The long-term annual mean temperature and precipitation are 17.07°C and 580 mm, respectively. The weather of this province is variable and is classified as a region with a semi-arid climatic condition (16). 

### Sample collection

This case-control study was performed on 240 serum samples from women with first spontaneous abortion referred to the only maternity hospital in Khorramabad city, during 2016, as the case group. The control group consisted of 240 serum samples from women who had a normal delivery and referred to the hospital for a checkup and had no history of abortion. All of the subjects in both the case and control groups had a history of at least one successful pregnancy, as those who did not have successful pregnancies were not included in the study. After obtaining the written consent from the participants in the study, a questionnaire based on age, education (Low literate, Diploma, Academic degree), occupation (Employee, Student, Housewife), place of residence (Urban, Rural), contact with cats, and consumption of raw/half-cocked meat was filled out by the participants. The blood sampling and serum isolation procedures were done under sterile conditions.

### ELISA

The level of anti-T. gondii IgM and IgG antibodies were measured in serum samples using the commercial kit, de EIA de *Toxoplasma* IgG Foresight® ACON, according to the manufacturer’s instructions (17). All specimens were run in duplicates. The results were considered positive when OD450 index was equal or higher than the cut off value. The cut-off values are estimated using known independent negative sera which are included in the titer-plates amongst the unknown samples.

### Avidity ELISA

To distinguish between the acute and chronic infections, all IgM- and IgG-positive samples of the case group were examined to evaluate IgG avidity by using the ELISA kit according to the manufacturer.s instruction (ELISA: Euro immune Kit, Germany). The test result is expressed as relative index avidity (RIA). According to the kit manual, the values less than 40% were considered as negative while the value more than 60% were considered positive and the borderline ranged between 40-60% (18). 

### Statistical analysis

Statistical analysis was done using the SPSS 22.0 software (SPSS Inc., Chicago, IL, USA). The Logistic regression and chi-square tests were used to evaluate the association between the *T. gondii* seropositivity and potential risk factors. Differences were considered significant when the P<0.05.

### Ethical statement

This study was approved by The Ethics Committee of Lorestan University of Medical Sciences (No. 200.93.11707). The written informed consent was obtained from all the participants before sampling. 

## Results

### Serology status and demographic information 

The results of the *Toxoplasma* serology status of participants in the study are shown in Table 1. The mean age was 27.01 ± 6.459 in the control group and 27 ± 6.499 in the case group. The mean of parity and gravidity of the control group, because of a lack of abortion history, were the same and it was 1.71 ± 0.86. The mean of parity and gravidity of the case group were 0.88 ± 0.99 and 1.88 ± 0.99, respectively. Our results, no significant association was seen between the maternal age and abortion (P=0.989). The results showed that the seropositivity rate for Toxoplasma IgM in the samples of the case group was 3.3% (8/240), while in the control group it was only 0.4% (1/240) of the samples, leading to a statistically significant difference between the two groups (P=0.019). The positive anti-*T.* gondii IgM antibodies had an odds ratio of 10.266, suggesting that the risk of abortion among women with positive IgM was about ten times higher than the other cases (P=0.019). Also, 47.5% (114/240) of the case group and 46.3% (111/240) of the control group were positive for anti-*T.* gondii IgG antibodies, but there was no statistically significant difference between two groups (P=0.784). 

Additionally, there was no significant difference between the case and control groups in terms of the prevalence of abortion in relation to education level (P=0.645) or the place of residence (city versus rural areas) (P=0.404). Out of all participants, 75.8% (182/240) of the case group and 72.5% (58/240) of the control group were living in the city. The results also showed that most of the women who had an abortion (67.1%) were housewives, and most of the women in the control group (61.7%) were employees, indicating that there is a significant difference in the relationship between occupation status and abortion rate (P<0.001). Also, 15% (36/240) of the women in the case group and 13.8% (33/240) of the control group kept a cat at home, but there was no significant difference between the two groups with regards to living near a cat (P=0.39).

**Table 1:** Compare of seroprevalence of toxoplasmosis between women with first spontaneous abortion and control group


Variable		Case group n=240	Control group n=240	P value

Age (Y)		27 ± 6.499	27.01 ± 6.459	0.989
Level of education				0.645
	Low literate	73 (30.4)	69 (28.8)	
	Diploma	109 (45.4)	104 (43.3)	
	Academic degree	58 (242)	67 (27.9)	
Occupation				<0.001
	Employee	168 (70)	79 (32.9)	
	Housewife	72 (30)	161 (67.1)	
Residence in the city				0.404
	Urban	182 (75.8)	174 (72.5)	
	Rural	58 (24.2)	66 (27.5)	
Contact with cats				0.696
	Yes	36 (15)	33 (13.8)	
	No	204 (85)	207 (86.2)	
Seropositivity rate for *Toxoplasma* IgM				0.019
	Yes	8 (3.3)	1 (0.4)	
	No	232 (96.7)	239 (99.6)	
Seropositivity rate for *Toxoplasma* IgG				0.784
	Yes	114 (47.5)	111 (46.3)	
	No	126 (52.5)	129 (53.7)	


Data are presented as mean ± SD or n (%).

### Avidity ELISA

All samples, which were positive in terms of anti-Toxoplasma IgM in both groups (9 samples) and IgG in the case group (114 samples), were evaluated by IgG avidity. Seven out of 8 (87.5%) sera, which were related to the case group, had low avidity indicating acute infection, whereas all positive IgG sera (100%) and 1 positive IgM sample, which was related to the control group had high avidity indicating chronic infection. 

## Discussion

Maternal acute toxoplasmosis or congenital toxoplasmosis during pregnancy is one of the important factors that increase the chance of abortion. It was previously believed that the congenital toxoplasmosis is due to an initial infection that occurs during pregnancy (13), but not to the reactivation of a latent infection in pregnant women with an immune deficiency (19). In addition, some believe that latent toxoplasmosis can be reactivated to cause the congenital transmission of parasites to their fetus (20). Serological evidence suggests a high prevalence of toxoplasmosis worldwide (21), and in fact, based on several studies Iran is one of the countries with a considerable prevalence (9, 13, 22). 

In this survey, we found that 8 out of 240 cases had the *T. gondii*-specific IgM antibodies, while in the control group there was only 1 woman with a positive result for anti-*T. gondii* IgM antibody. This observation may indicate a significant relationship between spontaneous abortion and acute toxoplasmosis. Also, our results showed that 47.5% of the case and 46.3% of the control group were positive in terms of anti-Toxoplasma IgG antibodies. There was no statistically significant difference between the two groups for *Toxoplasma*-specific IgG antibody, which is consistent with the results of a number of studies, yet, inconsistent with a few others. In a research project that was conducted in Bandar Abbas, Southern Iran, 124 women with an abortion history were studied for the frequency of anti- Toxoplasma IgG and IgM antibodies. The results showed that 79.03% and 15.32% of those women were positive for anti-*Toxoplasma* IgG and IgM antibodies, respectively (12). Also, a meta-analysis study on the relationship between toxoplasmosis and its outcomes showed that the infection rate in the abnormal-pregnancy group was significantly higher than the normal-pregnancy group (23).

With regards to other issues that may play as risk factors for abortion, our results showed that there was no significant relationship between the rate of abortion and urban or rural residence. Based on the results of this study with those of the present research, it can be concluded that health education and training classes for villagers has been effective in increasing the level of knowledge and personal care. Although there was no significant relationship between the level of education and prevalence of abortion, a significant correlation was observed between having a job and prevalence of abortion, since in the case group, 67.1% of the IgG-positive cases were housewives, and in the control group, 61.7% of the women were employees. The reason can be referred to as the lifestyle, which can affect the level of information. Furthermore, according to our results, there was no relationship between keeping a cat at home and the rate of abortion (24). In a study conducted in Egypt, the results showed that the seroprevalence of toxoplasmosis in high-risk pregnancy group was significantly more than a normal pregnancy group. Also, not consistent with our findings, in their study there was a significant difference between seropositivity and both living in a rural area, and undercooked meat consumption (25).

Due to the wide range of clinical signs of toxoplasmosis and the chance of getting confused with other diseases, it is necessary to use laboratory methods to confirm the clinical diagnosis. The serological assays have different sensitivities and specificities and are based on the affinity and avidity of antibodies. Detection of specific IgG antibodies is rarely a problem and has good sensitivity and specificity in different methods (26). In contrast, isolation and detection of IgM antibodies due to the less specificity of the methods used, long-term half-life or false IgM antibody resulted from other infections, leads to false-positive results, unnecessary treatments and even a false decision to terminate the pregnancy (27). Some IgM kits have lower reliability and credibility, which leads to an unacceptable increase in false-positive test results (28). For this purpose, in 1997, the Food and Drug Administration (FDA) advised physicians in the USA to clarify these limitations and advised laboratory staff and physicians to make sure of the quality of the kits prior to making decisions about clinical management of patients (29). For this purpose, the FDA has recommended that the positive IgMs should be approved by IgG avidity test, which is provided to discriminate between the old and new *Toxoplasma* infections (30), that is highly important in pregnant women and people with immune deficiencies (31). This method, primarily developed by Hedman et al. (32) in Finland, is now available as a kit throughout the world. The binding strength of IgG to* T. gondii* antigen shifts from low avidity to high within 5 months. This is the means by which it is possible to differentiate a recent infection from an old one in the first trimester of pregnancy in women with IgM or IgG (33).

In this study, IgG avidity test was used to evaluate IgM- and IgG-positive cases. The result showed that 8 cases of IgM-positive in the case group had low avidity, which indicated an acute phase of infection. On the other hand, 1 case of IgM-positive in the control group had high avidity, indicating a chronic phase of infection. Disagreement in the results of different studies may be due to the patients’ varying health status, differences in consumption of raw or undercooked meat, and keeping cats as pets at home (34). In the current study, eating roast meat (locally called Kebab) was very common, as it is a traditional food with a high rate of consumption. As a result, this eating habit is one of the important risk factors for *Toxoplasma* infection. Consuming well-cooked Kebab could be considered as a way of reducing the risk factor and preventing *T. gondii* infection in our population. Interestingly, the eating habits of the people as well as the climate of this region has led to several research studies on the prevalence and treatment of other parasitic infections in Lorestan province (35-40).

Healthcare providers in women’s hospitals should know that in the case of a pregnant woman the avidity test is not a validated and final test to be used solely for decision making, and that an equivocal IgG avidity result should not be considered in the diagnosis process.

## Conclusion

According to the findings of this study, it is suggested that the necessary health information, especially on the *Toxoplasma* transmission routes to women before marriage, particularly for the seronegative women, be provided and easily available. Additionally, indicating the sensitivity of a woman to acute toxoplasmosis, as well as the serological assessment of toxoplasmosis, before and during pregnancy, is recommended. Although sometimes these assays do not lead to a definitive interpretation, more sensitive methods such as amniotic fluid studies using molecular techniques are also needed to decide on treatment or termination of a pregnancy. In addition, the PCR and anti-*T. gondii* IgG avidity assays should be performed in medical diagnostic laboratories for accurate identification of the initial infection of toxoplasmosis in pregnant women.
